# Dentifrice

**Published:** 1858-01

**Authors:** 


*Dentifrice.*—Dr. Cramoisy recommends a powder composed of 1543 grains of carbonate of lime, and the same quantity of calcined magnesia, with 30 drops of essence of mint, as a dentifrice.

